# Pediatric Sleep Questionnaire Predicts Moderate-to-Severe Obstructive Sleep Apnea in Children and Adolescents with Obesity

**DOI:** 10.3390/children9091303

**Published:** 2022-08-27

**Authors:** Giuseppina Rosaria Umano, Giulia Rondinelli, Margherita Luciano, Alessandro Pennarella, Francesca Aiello, Giuseppe Salvatore R. C. Mangoni di Santo Stefano, Anna Di Sessa, Pierluigi Marzuillo, Alfonso Papparella, Emanuele Miraglia del Giudice

**Affiliations:** Department of the Woman, the Child, and General and Specialized Surgery, University of Campania Luigi Vanvitelli, 80138 Naples, Italy

**Keywords:** obesity, children and adolescents, pediatric sleep questionnaire, OSA, screening

## Abstract

Pediatric obesity is associated with an increased risk of morbidity during childhood. Alongside the well-known metabolic syndrome, during the last decades scientific research has deeply investigated the risk of sleep breathing disorders. Among them, obstructive sleep apnea (OSA) commonly affects children with obesity. The presence of OSA heightens the risk of metabolic impairment and weight gain. Therefore, it deserves specific treatment. However, polysomnography (PSG) is not always available in clinical settings, and alternative diagnostic tools are needed. This study aimed to investigate the predictivity of the pediatric sleep questionnaire (PSQ) for moderate-to-severe OSA diagnosis. Children and adolescents with obesity and suspected OSA with available full-night cardiorespiratory PSG were retrospectively enrolled. Receiver operating curve analysis was performed to test the ability of PSQ in predicting moderate-to-severe OSA (AHI > 5 episode/h). The final sample included 60 children and adolescents. The PSQ showed a good area under the curve (AUC) of 0.88 (95% CI 0.78–0.98, *p* < 0.0001). Moreover, a value above or equal to 0.65 showed an 80% sensitivity and 100% specificity for moderate and severe OSA. These findings suggest that PSQ might be used in clinical settings with limited access to PSG for stratifying disease severity and for selecting children with urgent need of sleep study.

## 1. Introduction

Obesity is a multifactorial condition defined as an abnormal or excessive fat accumulation with an increased risk of morbidity and mortality [1]. During the last decades, the prevalence of obesity in children and adolescents aged 5–19 years increased from 4 to 18% leading to the creation of a new term: “globesity”. An excessive amount of adipose tissue is the main risk factor for the development of metabolic syndrome and other obesity-related comorbidities, such as cardiovascular accidents [2], hypertension [3], dyslipidemia [4], polycystic ovary syndrome (PCOS) [5], non-alcoholic fatty liver disease (NAFLD) [6], and respiratory diseases, even during childhood. There is a strict connection between obesity and sleep disorders, particularly obstructive sleep apnea syndrome (OSA) [7].

OSA is a disorder caused by repetitive upper airway collapse during sleep, with subsequent oxyhemoglobin desaturations and sleep fragmentation. Consequently, OSA causes sleep disruption, cognitive deterioration, and increased low-grade inflammation [8]. Recent studies showed that about 1 out 2 subjects with OSA are obese and it is also estimated that, in severe obesity, the prevalence of sleep apnea ranges between 40% and 90% [9]. Concerning pediatric age, OSA prevalence ranges between 1–4% [10], and these rates rise to 60% in children and adolescents with obesity [11].

Although obesity represents one of the main risk factors for OSA, it, in turn, can cause weight gain and increased risk of metabolic syndrome compared to children and adolescents without OSA [12,13]. The reasons for this phenomenon are to be found in reduced physical activity, insulin resistance, and increased ghrelin levels. The evidence suggests that the association between OSA and obesity promotes further weight gain and metabolic impairment creating multiple vicious cycles [14]. Therefore, the diagnosis of OSA in obese patients represents a matter of primary importance.

OSA diagnosis is performed by polysomnography (PSG), a multi-parameter sleep study performed overnight [12]. PSG allows the contemporary investigation of several body functions, including brain activity, eye movements, skeletal muscle activation, respiratory muscle effort, oral-nasal airflow, heart rhythm, and oximetry. OSA severity is scored as the sum of obstructive events (both apneas and hypopneas) divided by the number of hours of sleep (apnea-hypopnea index, AHI). An AHI > 1 is indicative of OSA, mild OSA is diagnosed for 1 < AHI ≤ 5, moderate OSA for 5 > AHI < 10, severe OSA for AHI ≥ 10. However, PSG is limited by some disadvantages such as patient inconvenience, foreign sleep environment, hospitalization, and economic costs due to highly trained staff and technology involved [12]. Therefore, more feasible tools for OSA diagnosis and screening have been developed and approved for settings with limited resources. Moreover, predictors of moderate and severe OSA forms might serve as triaging systems for waiting list management in sleep centers [12]. Among them, the Pediatric Sleep Questionnaire (PSQ) is a validated screening method [15,16,17]. This parent-reported questionnaire contains 22 items about snoring frequency, loud snoring, observed apneas, difficulty breathing during sleep, daytime sleepiness, inattentive or hyperactive behavior, and other features [18]. The responses are coded as “yes” = 1, “no” = 0, and “don’t know” = missing (Table 1). PSQ score is calculated as the ratio between the sum of yes and number of answered questions; a PSQ score ≥ 0.33 is indicative of OSA. Other questionnaires are available, and some of them have been associated with moderate-to-severe OSA in children. To date, there are no data available on PSQ values indicating severe OSA. Based on this knowledge gap, this study aims to verify the association of PSQ with polysomnographic characteristics in children and adolescents with obesity and to identify a predictive cut-off for severe OSA.

Adapted from the original version of the Pediatric Sleep Questionnaire.

## 2. Materials and Methods

We retrospectively enrolled children and adolescents with obesity attending the pediatric unit of the University of Campania “Luigi Vanvitelli” from January 2017 to December 2020 because of obesity and nocturnal snoring. Children were eligible if they had obesity (i.e., a body mass index (BMI) ≥ 95th for age and sex according to reference charts) [19] and presented a pathological score on a sleep questionnaire for OSA screening [15].

Exclusion criteria included drug assumption and secondary forms of obesity. Parents and children were asked to sign a written informed consent before any procedure.

The study was conducted according to the criteria set by the Declaration of Helsinki and was approved by the local ethical committee (protocol n. 834/2016). All subjects underwent an anthropometric evaluation and overnight cardiorespiratory polygraphy.

### 2.1. Clinical Examination

Weight was measured by a balance beam scale; the child being undressed. Height was measured by a Harpenden stadiometer. BMI and Z-score BMI were calculated with the lamba-mu-sigma method [20] according to reference charts [19].

### 2.2. Pediatric Sleep Questionnaire

The Pediatric Sleep Questionnaire (PSQ) scores 22 items that investigate presence and intensity of snoring, presence of obstructive apneas and breathing difficulties, sleepiness, and other symptoms that correlate with pediatric OSA. PSQ has been validated for OSA screening with cardiorespiratory polygraphy in children [15]. Each item is scored as being present, absent, or unknown. The score is obtained as the following ratio for answered questions: present/(present + absent); any questions that are not answered (unknown) are not included in the calculation. A cut-off value of 0.33 indicates suspected OSA [15,16].

In this study, all children and adolescents presenting a PSQ score ≥ 0.33 underwent a full night cardiorespiratory polygraphy.

### 2.3. Cardiorespiratory Polygraphy

Sleep recordings were performed with Embletta^®^ Gold (Embla Systems Inc., Kanata, ON, Canada). They included nasal airflow (pressure), oral airflow (thermistor), oximetry, respiratory thoracic and abdominal muscles activity (inductance plethysmography), and electrocardiogram. American Academy of Sleep Medicine (AASM) scoring criteria 2012 for children were used for records analysis [21]. Obstructive events including apnea and hypopnea were scored according to AASM criteria. The final apnea-hypopnea index (AHI) was determined by dividing the total number of obstructive apneas and hypopneas by the hours of sleep. An AHI of >1 was diagnostic for OSA. Other respiratory parameters recorded for the analysis were: mean oxygen saturation, oxygen desaturation index (ODI), lowest oxygen saturation (Nadir), and mean oxygen desaturation. OSA severity was scored as: mild OSA for 1 < AHI ≤ 5, moderate OSA 5 > AHI < 10, and severe OSA AHI ≥ 10 [21].

Cardiorespiratory polygraphy, even if does not include electroencephalography, is sufficiently accurate for OSA diagnosis [10].

### 2.4. Statistical Analysis

Normal distribution for continuous variables was investigated by Kolmogorov-Smirnov test. Kruskal-Wallis test was performed to check differences in continuous variables. Post-hoc analysis for multiple comparisons was performed with the Dunn test.

A Chi-square test was performed for differences in categorical variables. Spearman correlation analysis was performed to test the correlation between PSQ and AHI. The predictive power of the PSQ score for severe OSA was determined with the receiver operating characteristic (ROC) curve test. The best predictor cut-off value was obtained using the Youden index (maximum (sensitivity + specificity − 1)). Data are expressed as median (interquartile range, IQR) or frequencies. All the analyses have been performed using SAS^®^ on Demand for Academics (SAS Institute Inc., Cary, NC, USA).

## 3. Results

The cohort included 60 children and adolescents (32 males) with a mean age of 11.4 ± 2.8 SD and a mean Z-score BMI of 3.05 ± 0.82 SD. The clinical and polysomnographic characteristics are depicted in Table 2.

OSA prevalence was 91.2, 41.1% of children had mild OSA, 27.9% moderate OSA, and 22.8% severe OSA. The mean PSQ score was 0.60 ± 0.16. PSQ values showed good correlation with AHI (r = 0.73, *p* < 0.0001). Difference between groups according to OSA severity are reported in Table 3. The groups did not differ for age, Z-score BMI, and sex distribution. Conversely, significant differences were found for PSG parameters and PSQ score (Table 3).

**Figure 1:**
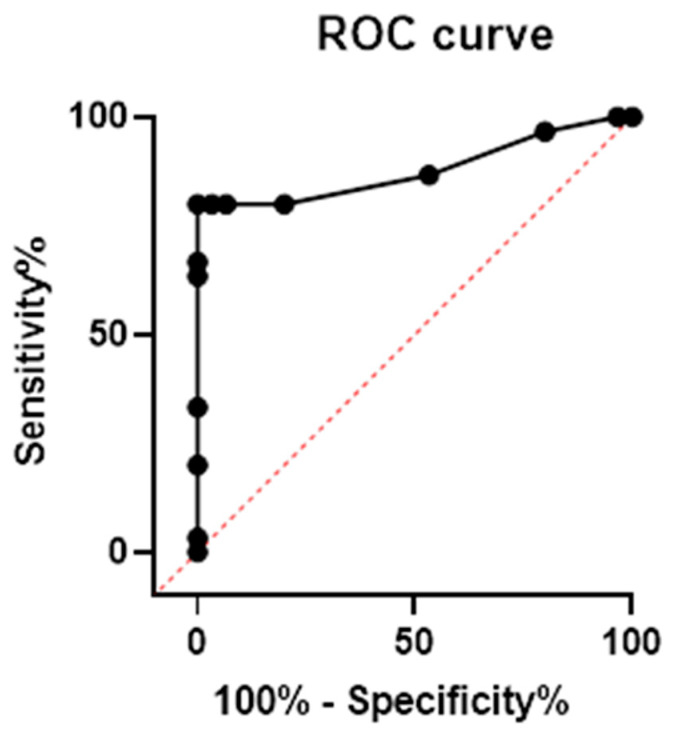
**Receiver operating characteristic Curve analysis of PSQ for diagnosis of moderate-to severe OSA.** PSQ showed a good accuracy in predicting moderate-to-severe OSA with an AUC of 0.879 (95% CI 0.779–0.975, *p* < 0.0001). The cut-off of 0.65 showed a sensitivity of 80% and a specificity of 100%. Legend: ROC: receiver operating characteristic.

ROC curve analysis showed that PSQ had good accuracy for moderate-to-severe OSA diagnosis, with an AUC of 0.879 (95% CI 0.779–0.975) and a *p* < 0.0001 (Figure 1). Then we performed the Youden test to calculate a specific PSQ score cut-off. Based on its ROC coordinates, the optimum value for detecting moderate-to-severe OSAS was 0.65, corresponding to a sensitivity of 80% and a specificity of 100%.

## 4. Discussion

PSQ is a recognized tool for identifying sleep disorders in the pediatric population. As mentioned above, a PSQ value greater than or equal to 0.33 is predictive of OSA (AHI > 1 episodes/h) with good accuracy. Moreover, the PSQ showed a good correlation with AHI. In this retrospective study, the efficacy of PSQ in predicting severe OSA in children and adolescents with obesity in comparison with PSG was evaluated. The cut-off value of 0.65 showed the best sensitivity (80%) and specificity (100%) for moderate-to-severe OSA in children and adolescents with obesity. It is of particular importance to pose the diagnosis of moderate-to-severe OSA as these forms deserve treatment in light of disease persistence and potential comorbidity. Therefore, in settings where PSG is unavailable, PSQ can be used to exclude moderate-to-severe OSA diagnosis. Additionally, in settings where many patients need PSG study, PSQ might be used to triage disease severity and give priority to those at higher risk for more severe disease.

Over the last decades, several screening questionnaires for pediatric OSA screening have been produced with heterogeneous results [22,23,24,25]. The OSA-18 questionnaire displays a sensitivity of 94% and 55% specificity for OSA diagnosis. However, it is not accurate in stratifying disease severity [25]. Chan et al. [26] have evaluated a modified Epworth Sleepiness Scale (ESS) and found that an ESS score > 8 could predict moderate or severe OSA with a very low sensitivity of 29% and a high specificity of 91%.

In fact, several studies have described the inaccuracy of anamnesis alone in detecting OSA and distinguishing between primary snoring and other sleep breathing disorders [22,27]. Montgomery-Downs et al. reported that a parent-report questionnaire was able in identifying sleep breathing disorders but not in distinguish between primary snoring and OSA [22]. Instead, Goldstein et al. [23] validated a clinical assessment score (CAS-15) based on a questionnaire (OSA-18) and physical examination against PSG for OSA diagnosis (i.e., AHI > 2). A CAS-15 score ≥ 32 had a sensitivity of 77.3% and a specificity of 60.7% for OSA. Similarly, the sleep clinical record (SCR) proposed by Villa and colleagues is useful for OSA screening [28], and an SCR score above 8.25 is indicative of moderate-to-severe OSA with a sensitivity of 83% and a specificity of 70% [24].

It is essential to identify children with severe OSA even if there are not enough resources to perform PSG considering the OSA-related comorbidities and the need for treatment. Moreover, this might be a useful tool in pre-operative settings for anesthesiologists and sedation physicians in patients undergoing surgery without a previous PSG to determine if a patient is appropriate for an outpatient surgery center or what to expect their degree of post-anesthetic airway obstruction to be. Additionally, children and adolescents with obesity represent a category at higher risk of showing OSA and metabolic impairment. Accurate and feasible screening tools able to stratify disease severity are advocated in clinical practice, and rapid sleep questionnaires are the most intriguing instruments.

This study has limitations that should be acknowledged. The major limitation is the use of cardio-respiratory PSG instead of complete PSG, which might underestimate the severity of the diagnosis. In addition, the study population includes children and adolescents with obesity, a category at higher risk for OSA, and this is the major limitation. Moreover, the age range does not include pre-school children. Additionally, the small sample size also might limit the generalizability of these findings to the general population. However, children with obesity represent a high percentage of children with OSA and might present OSA-related comorbidities already at diagnosis.

## 5. Conclusions

This study provides the identification of a PSQ cut-off value for moderate-to-severe OSA in children and adolescents with obesity. The cut-off shows good sensitivity and specificity and might be used in clinical settings to triage patients with suspected OSA. In consideration of economic costs and the poor availability of PSG in many settings, the implementation of feasible and accurate screening tools should be advocated. The PSQ is a better predictor of moderate or severe OSA compared to other questionnaires that have been previously evaluated. The comparison of PSQ with a composed score, such as SCR and CAS-15, should be investigated in children and adolescents with obesity. Additionally, this cut-off should be confirmed in other larger pediatric cohorts.

## Figures and Tables

**Table 1:** Pediatric sleep questionnaire items [15].

Questions
Does he/she snore more than half the night?
Does he/she often snore?
Does he/she snore noisily?
Does he/she breathe loudly or heavily?
Does he/she have trouble breathing, or effort to breathe?
Have you ever seen your child stop breathing?
Does he/she breathe out of his/her mouth during the day?
Does he/she have a dry mouth when awake in the morning?
Does he/she wet the bed?
Does he/she awake in the morning without being refreshed?
Does he/she have trouble with sleepiness in the daytime?
Have a teacher or another supervisor noticed that he/she seems to be asleep during the daytime?
Is he/she difficult to awaken in the morning?
Does he/she awake in the morning with headaches?
Has he/she ceased growing normally since birth?
Is he/she overweight?
He/she doesn’t appear to listen when you talk to him/her directly
He/she has trouble organizing duties and activities
He/she is easily distracted by foreign stimulation
He/she violins with hands or feet, or twitching in seating
He/she is “on the move” or often acts like he/she is “powered by an engine”
He/she interrupts/disturbs others (i.e., interferes with conversations/games)

**Table 2:** Anthropometric and polysomnographic parameters of the cohort.

Parameter	Value
Sex (M, %)	54.1
Age	11 (9.2–14)
Weight	79.8 (60.9–103)
Height	1.5 (1.4–1.6)
BMI	32.6 (25.5–37.1)
BMI z-score	2.9 (2.5–3.4)
Waist-to-Height ratio	0.65 (0.59–0.7)
OSA (PS/mild/moderate/severe, %)	8.2/41.1/27.9/22.8
PSQ	0.52 (0.45–0.77)
AHI	5.0 (2.9–8.5)

Data are expressed as median (interquartile range) and frequencies. Legend: AHI: apnea-hypopnea index; OSA: obstructive sleep apnea; PS: primary snoring; PSQ: pediatric sleep questionnaire.

**Table 3:** Differences according to OSA group.

	Primary Snoring (*n* = 5)	Mild (*n* = 26)	Moderate (*n* = 18)	Severe (*n* = 12)	*p*
Age	13.9 (13.6–14.7)	11 (9.2–14.2)	12.1 (9.8–13.6)	9.9 (8.4–11.9)	0.16
ZS-BMI	2.99 (2.5–3.2)	3.1 (2.4–14.1)	2.9 (2.5–3.2)	2.9 (2.6–3.7)	0.57
Sex (M, %)	40.0	46.1	50.0	66.6	0.11
PSQ	0.45 (0.36–0.46)	0.50 (0.45–0.51)	0.68 (0.47–0.77)	0.82 (0.82–0.86)	**<0.0001**
AHI	0.9 (0.8–0.9)	3.2 (2.5–3.9)	6.7 (5.8–7.5)	24.1 (20.3–39.7)	**<0.0001**
ODI	0.4 (0.4–0.4)	2.1 (1.4–3.4)	5.2 (3.3–6.8)	26 (18.7–37)	**<0.0001**
NADIR	93 (93–94)	92 (89–92)	90 (89–92)	78 (67–84)	**<0.0001**
Mean SpO2	97.6 (96.9–97.7)	96.7(96.1–97.6)	97 (96.2–97.4)	95 (91.3–95.8)	**0.002**
Mean desaturation	3.8 (3.1–3.8)	3.7 (3.5–4.1)	3.9 (3.6–4.1)	5.3 (4.6–7.4)	**0.001**

Data are expressed as median (interquartile range) and frequencies. Statistically significant *P* values are reported in bold. Legend: AHI: apnea-hypopnea index; ODI: oxygen desaturation index; OSA: obstructive sleep apnea; PSQ: pediatric sleep questionnaire, SpO2: peripheral capillary oxygen saturation.

## Data Availability

Data are available upon motivated request to corresponding author.
